# Surgical Diseases of the Tongue

**Published:** 1887-02

**Authors:** H. A. Smith

**Affiliations:** Cincinnati, Ohio.


ARTICLE IV.

### SURGICAL DISEASES OF THE TONGUE.

BY H. A. SMITH, D. D. S., CINCINNATI, OHIO.

All along down from the first records of medicine the appearance of the tongue have been studied in connection with morbid conditions of the system. Its appearance in health and disease, it properly studied, would often enable us to advise our patients as to whether the peculiar appearances presented are due to local conditions present in the mouth or to sympathy with the alimentary canal, or perhaps with other parts of the system.

The tongue, situated as it is in the floor of the mouth, is surrounded by the teeth. These, if healthy and intact, form a protecting wall to shield it from harm, but if diseased or broken down they become the veritable cause of a variety of diseases of the tongue.

In this connection we may mention *parasitic affections* of the tongue. While animal parasites are rarely found on the tongue, it may safely be said that no tongue, however healthy, is free from *vegetable* parasites; and yet Butlin states that there is but one disease, so far as he knows, due to the presence of a vegetable parasite, namely *thrush*, a membranous disease not only of the tongue, but of the inside of the mouth generally. The disease depends upon the presence of a fungus (*oidium albicans*), which is identical with the oidium lactis, the ferment of the acid fermenta tion of milk.

These two parasites being morphologically alike, we should expect to find in the mouths of children who are attacked with thrush after the teeth begin to erupt that caries would be more prevalent with those who are fed with a spoon than those fed with mother's milk. In children artifically fed the mouth shows more constantly an acid reaction. In the act of sucking the saliva is secreted in larger quantities, and being alkaline, the acidity of the fluids of the mouth neutralized to a degree not found in children fed with a spoon.

The condition called *furred tongue* was formerly regarded as nearly always a sign of disease; variation in the extent and color of the coating indicating the particular form of disease.

Greater weight must be given these appearances in diagnosis of disease if we consider this deposit of fur as proceeding from a secretory process of tongue itself (Wood) than when we accept the modern view that fur on the dorsum of the tongue, whether in health or disease, is essentially a growth of fungus (chiefly of micrococcus and bacillus subtilis), and that the epithelium and food debris usually present are unimportant and accidental constituents (Butlin).

The vegetable spores found in the fur are deposited in the filiform papillæ of the tongue from the food taken in the mouth, as well as from the inspired air, and finding a favorable habitat the germ grows with surprising rapidity especially at night when the tongue is comparatively still.

Frequently the tongue is prevented from being cleansed in the natural way by the absence of one or more teeth on the side of the mouth, by the roughened surface of a tooth, or the presence of diseased teeth that are sore to the touch, thus preventing sufficient movement and friction against the teeth to cleanse the tongue. If a person eats on one side of the mouth, the tongue on the opposite side will show more or less furring. Mr. Hilton attributes this furring of the tongue to disturbance of function and nutrition by reflex action through the influence of the fifth nerve. But, as Mr. Hutchinson remarks, most of these examples of unilateral furring may be explained by mechanical action.

The tongue is subject to quite a variety of ulcers. Of the traumatic variety, those that are caused by the teeth wounding or irritating the tongue frequently come under the care of the dentist. Unless the patient is subject to chronic glossitis, these simple ulcers are speedily cured by removal of the cause of the disturbance, a very simple operation frequently, as by carefully smoothing the jagged edge of the tooth or by extraction.

Ulcers which form on the under side of the tongues of children attacked by whooping cough, and which are supposed to be a specific lesion of the disease, may be, we think, explained by the irritation caused by the rubbing of the tongue against the lower teeth during the paroxysms of coughing.

Aphthous ulcers are frequently met with upon the tongue and mucous membrane of the mouths of adults, but they are more common in children; and it is still a moot question whether or not the disease is contagious. If a specific parasite is always present in aphthous ulceration, then it is possible for a dentist, unless proper antiseptic precautions are observed, to convey the pathogenic germ from an aphthous patient for whom he has operated to the mouth of another susceptible patient.

Among the exciting causes of *cancer* of the tongue usually mentioned by writers are bad teeth, coarse, hot and highly spiced food; the constant friction of the mouth-piece in using a tobacco pipe, chewing tobacco, and the injudicious use of caustics in treating sores on the tongue (Butlin).

The relation of the disease to age and sex is somewhat striking. Statistics show that it is hardly known in young adult life, while it is quite common between the ages of 40 and 70.

We have mentioned the irritation caused by jagged edges and rough surfaces of the teeth as a cause of cancer of the tongue, and it may be noted in this connection that the teeth during the period of life between 40 and 70, if neglected, are most likely to take on the conditions tending to injure the tongue. To the hard usage and greater neglect to which the teeth of men are subjected, we may, in part, attribute the marked frequency of carcinoma in males over females. Baker, who gives a record of 293 cases, states that of these only 46 were females.

Another explanation of this difference in the liability of the two sexes to cancer of the tongue, is that women are not given to drinking, smoking and chewing, as are the men.

Carcinoma may attack any part of the tongue; but the fact that it is most frequently seated in the parts in contact with the teeth, emphasizes the importance of giving attention to these organs in diagnosis of this disease.

The question whether or not the habit of smoking predisposes to cancer of the tongue, is now frequently asked. Butlin says: "There is no evidence with which I am acquainted which will prove that carcinoma is really much more common among adult males who smoke than adult males who do not smoke." Yet I think it not improbable that smoking does to a certain extent predispose to the disease.

Diagnosis of lingual cancer is rendered quite difficult, because of the resemblance of syphilitic ulcerations, of tubercular ulcerations, of fissures of the tongue, and sometimes of simple ulcers, to carcinoma; and moreover, the difficulty is increased by the fact that certain of these diseases are transformed into carcinoma by almost imperceptible graduations.

In all doubtful cases the microscope is the most valuable and reliable aid to diagnosis.—*Dental Cosmos.*

